# Prospective randomized trial evaluating mandatory second look surgery with HIPEC and CRS vs. standard of care in patients at high risk of developing colorectal peritoneal metastases

**DOI:** 10.1186/1745-6215-11-62

**Published:** 2010-05-25

**Authors:** Robert T Ripley, Jeremy L Davis, Clinton D Kemp, Seth M Steinberg, Mary Ann Toomey, Itzhak Avital

**Affiliations:** 1 Surgery Branch, CCR, NCI, Bethesda, MD 20892-120, USA; 2 Biostatistics and Data Management Section, CCR, NCI, Bethesda, MD, USA

## Abstract

**Background:**

The standard of care for colorectal peritoneal carcinomatosis is evolving from chemotherapy to cytoreductive surgery (CRS) with hyperthermic intraperitoneal chemotherapy (HIPEC) for patients with disease limited to the peritoneum. Peritoneal carcinomatosis from colorectal cancer treated with chemotherapy alone results in median survival of 5 to 13 months, whereas CRS with HIPEC for early peritoneal carcinomatosis from colorectal cancer resulted in median survival of 48-63 months and 5 year survival of 51%.

Completeness of cytoreduction and limited disease are associated with longer survival, yet early peritoneal carcinomatosis is undetectable by conventional imaging. Exploratory laparotomy can successfully identify early disease, but this approach can only be justified in patients with high risk of peritoneal carcinomatosis. Historical data indicates that patients presenting with synchronous peritoneal carcinomatosis, ovarian metastases, perforated primary tumor, and emergency presentation with bleeding or obstructing lesions are at high risk of peritoneal carcinomatosis. Approximately 55% of these patient populations will develop peritoneal carcinomatosis. We hypothesize that performing a mandatory second look laparotomy with CRS and HIPEC for patients who are at high risk for developing peritoneal carcinomatosis from colorectal cancer will lead to improved survival as compared to patients who receive standard of care with routine surveillance.

**Methods/Design:**

This study is a prospective randomized trial designed to answer the question whether mandatory second look surgery with CRS and HIPEC will prolong overall survival compared to the standard of care in patients who are at high risk for developing peritoneal carcinomatosis from colorectal cancer (CRC). Patients with CRC at high risk for developing peritoneal carcinomatosis who underwent curative surgery and subsequently received standard of care adjuvant chemotherapy will be evaluated. The patients who remain without evidence of disease by imaging, physical examination, and tumor markers for 12 months after the primary operation will be randomized to mandatory second look surgery or standard-of-care surveillance. At laparotomy, CRS and HIPEC will be performed with intraperitoneal oxaliplatin with concurrent systemic 5-fluorouracil and leucovorin. Up to 100 patients will be enrolled to allow for 35 evaluable patients in each arm; accrual is expected to last 5 years.

**Trial Registration:**

ClinicalTrials.gov ID: NCT01095523

## Background

In the United States, approximately 108,070 patients are diagnosed with colon cancer and 40,740 patients with rectal cancer per year (colorectal cancer = CRC) [1]. 49,970 patients die from CRC per year. Initially, peritoneal carcinomatosis (PC) commonly occurs without systemic dissemination. In one study of the 3019 patients reviewed with CRC, 349 (13%) had carcinomatosis [2]. 214 (61%) patients had synchronous disease and 135 (39%) patients had metachronous disease. 58% of synchronous PC was limited to the peritoneal cavity and 64% of these patients had localized disease to one quadrant [2]. Overall, recurrences are limited to the peritoneum in 25% of patients with CRC [3]. Approximately 8000 patients are diagnosed with synchronous PC in the United States per year.

Modern chemotherapy has improved survival of patients with PC, but despite increased response rates and increased median survival, few patients experience long term survival with chemotherapy alone. The median survival of patients with PC without systemic dissemination was 7 months for the 3019 patients reported above [2]. Another study prospectively evaluated 45 patients with PC who achieved a median overall survival of 6 months [3]. 118 patients in a French registry showed a median overall survival of 5.2 months [4]. Most of these patients received systemic 5-FU and leucovorin-based regimens. Although multiple reports of metastatic disease demonstrate an increased survival with current chemotherapeutic regimens [5-10], Elias et al. reports the only series that follows patients with PC without systemic dissemination treated with current standard of care systemic chemotherapy regimens of FOLFOX (5-fluorouracil (5-FU), leucovorin, and oxaliplatin) and FOLFIRI (5-fluorouracil (5-FU), leucovorin, and irinotecan). The authors report a median survival of 23.9 months and 2-, and 5-year survival of 65% and 13%, respectively [11]. Clearly, patients have benefited from better systemic regimens, but at best, survival is only 13% at five years for this group of patients treated with modern systemic chemotherapy alone.

The prognosis of patients with PC has significantly improved with the combination of cytoreductive surgery (CRS) and hyperthermic intraperitoneal chemotherapy (HIPEC) [11-13]. The 5-year overall survival ranges from 40 - 51%. The technique has previously been described as peritonectomy, hyperthermia, and resection of intrabdominal disease as necessary [14-16]. Hyperthermia has been shown to increase the cytotoxicity of chemotherapy [17,18]. CRS and HIPEC initially began as the treatment for appendiceal malignancy and malignant peritoneal mesothelioma [19-21]. In 2006, this procedure was declared the standard of care by the National Cancer Institute for ovarian PC based on the results of a phase III study [22]. In the past 8 years, two randomized clinical trials (RCT), one nonrandomized comparative study, and 11 observational studies have been reported for colorectal carcinomatosis and summarized in a comprehensive review article by Yan et al [23].

Verwaal et al [24] performed a RCT of CRS and HIPEC versus systemic chemotherapy alone which demonstrated a significant survival benefit with increase in median survival of 22 mo. versus 12 mo. and 2-year survival of 44% versus 22%, respectively. Even though this trial demonstrates a clear benefit, the systemic therapy with 5-FU and leucovorin is an outdated regimen compared to current treatment of FOLFOX or FOLFIRI. In addition, the HIPEC drug was mitomycin-C which is not the most effective drug against CRC. Regardless, the patients who underwent intent-to-treat randomization and received CRS and HIPEC achieved a significant survival advantage (p = 0.032). For the patients who received an R0 resection, the 2-year survival was 60%. A multi-institutional registry [25] of 506 patients who underwent CRS and HIPEC for CRC with PC demonstrated a 1-, 3-, and 5-year survival of 72%, 39%, and 19%, respectively with a median survival of 19 months. Again, similar to the RCT data, patients with complete CRS achieved the highest survival. The patients who underwent R0 resection achieved a 1-, 3-, and 5-year survival of 87%, 47%, and 31%. The ten single institutional case-series reviewed by Yan et al. [23] demonstrated overall median survivals from 13 to 29 months and 1-, 2-, 3-and 5-year survival rates of 55-75%, 31-64%, 21-28%, and 11-19%, respectively. Clearly, with one trial of level 1 data, another with good level 2 data, and multiple case series reporting excellent survivals, CRS with HIPEC shows promise to become the standard of care for CRC with PC.

Unfortunately, PC is usually detected late in the course of disease secondary to late presentation of symptoms and difficulty with detection based upon imaging techniques and tumor markers. Once symptomatic disease develops, complete resection is more difficult to achieve. The only reliable means of detection is repeat laparotomy, but this operation cannot be justified in all patients with CRC. High risk group of patients who may benefit [26-28] have been defined after a prospective phase II trial as those patients with synchronous limited PC at initial operation, synchronous ovarian metastases, perforated primary tumors, T4 lesions that required adjacent organ resection, and emergency presentation for obstructing and bleeding lesions [26]. 62% of patients with limited PC at presentation, 75% of patients with ovarian metastases, 15% of patients with perforated CRC, and 19% of patients with T4 lesions that required resection of adjacent organs will develop PC [2].

Given the impact on survival with CRS and HIPEC, Elias et al. [26] performed a prospective study to analyze the outcomes with mandatory second-look surgery (MSLS) for patients at high-risk for recurrence as stated above. All patients were NED based upon symptoms, tumor markers, CT, and PET. Every patient had appropriate primary resection with adjuvant FOLFOX or FOLFIRI as the current standard of care. Six months after the last chemotherapy, MSLS was performed. Macroscopic PC was found in 55% of the patients (16/29) who were asymptomatic with negative evaluation. All of the patients with recurrence were treated with CRS and HIPEC. No post-operative mortality occurred. With a median follow-up of 27 months, of the 16 patients with recurrent peritoneal carcinomatosis, 8 (50%) remained NED, 4 (25%) relapsed with PC (2 with distant metastases), and 4 (25%) developed visceral metastases. Most importantly, this study revealed that high-risk patients with recurrent PC are often detected only at laparotomy despite appropriate surveillance and that the early detection of disease may result in complete CRS compared to CRS of symptomatic disease or disease detected by imaging.

Chemotherapeutic drugs used for intra-operative management are based on the most effective systemic regimens which also demonstrate good results regarding intraperitoneal delivery. Systemic oxaliplatin has demonstrated significant activity against metastatic CRC with an objective response rate of 24% as a single agent and 55% in combination with 5-FU and leucovorin (FOLFOX) [8,29,30]. FOLFOX is one of the first line standard of care regimens for metastatic CRC. Oxaliplatin is currently the most studied intraperitoneal drug [31-33] and it is also potentiated by hyperthermia and it works at all stages of cell division [17,34].

In summary, for approximately 50% of patients who develop PC from CRC, a complete resection may be potentially curative. Patients with high-risk primary CRC have an incidence of PC greater than 50%, yet modern imaging and tumor or serum markers fail to detect recurrence at an early stage. We hypothesize that MSLS with CRS and HIPEC in patients who are at high risk for recurrent PC will lead to improved survival compared to standard surveillance. Therefore, a prospective randomized trial has been designed to evaluate MSLS with CRS and HIPEC versus treatment of PC once disease is detected, during routine surveillance, by imaging or symptoms.

## Methods

### Ethical Approval

The Mandatory Second Look Surgery (MSLS) for high Risk Colorectal Cancer trial was approved by the Institutional Review Board (IRB) of the National Cancer Institute, National Institutes of Health, Bethesda, Maryland.

### Design

**Figure 1:**
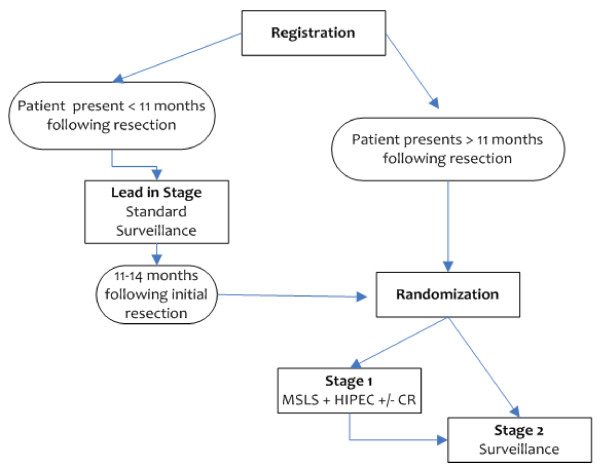
**Trial schema**.

This study is a randomized controlled trial. The trial schema is illustrated in Figure 1. The study will be performed at the Clinical Center of the NIH by the Surgery Branch of the NCI in Bethesda, Maryland, USA. Patients who have undergone curative surgical resection for the primary CRC tumor, are at high risk for development of PC, have completed at least 3 months of standard of care systemic therapy, and show no evidence of disease may be enrolled and randomized to receive either standard of care surveillance (SC) or mandatory second-look surgery (MSLS) followed by CRS and HIPEC. Randomization will occur between 11 - 14 months after the primary operation to allow flexibility in enrollment and accrual without effect on design.

### Stratification and Randomization

Registration of patients onto this study will take place within 24 hours of the patient signing the consent by faxing a completed eligibility checklist to the Central Registration Office. This trial will consist of three stages: Lead-in Stage includes the time period from 3 months to 14 months following resection. Patients who present for evaluation prior to month 11 post resection will be enrolled in this stage prior to randomization. Stage 1 includes MSLS with HIPEC and CRS and the recovery period. Patients who are randomized to receive surgical intervention will be entered on stage 1. Stage 2 is the follow up period. Patients who are randomized to surveillance will be directly entered on stage 2; patients who are randomized to the MSLS arm will be entered on stage 2 upon recovery from the operative procedure. After 11-14 months following primary resection, the patients will be stratified and randomized by the Central Registration Office to undergo MSLS with HIPEC and CRS or standard-of-care surveillance (SC) without surgery. The Central Registration Office will notify the study coordinator of the results of randomization. Patients must be randomized within 2 weeks of the baseline imaging studies. Patients who are randomized to the MSLS arm must be treated within 2 weeks of randomization.

Patients will be stratified according to two factors: 1: Presenting with PC, which was completely resected, and/or ovarian metastases (incidence of recurrent PC > 60%) vs. presenting with tumor perforation, T4 lesions that required adjacent organ resection, and/or emergency presentation with bleeding or obstructing lesions (incidence of recurrent PC < 40%), and 2: Receiving adjuvant chemotherapy for less than 6 months vs. > 6 months. This will generate 4 strata.

### Statistics

The primary objective of the trial is to determine if there is a difference in overall survival among patients who are at high risk for CRC PC and are randomized to receive either SC alone or MSLS with HIPEC and CRS when indicated.

Based upon results in the literature, patients who would be eligible to be randomized on this trial and who receive SC alone would be expected to have an estimated 25% 5-year survival from the date of randomization. The goal of this study will be to determine if the use of a MSLS with HIPEC and CRS will result in patients having survival that is associated with an increase to 50% 5-year survival. Following registration, patients who are eligible for randomization will be randomized between SC and MSLS with HIPEC and CRS and followed for survival. Patients will be stratified for synchronous peritoneal carcinomatosis and ovarian metastasis vs. perforated primary and emergency with bleeding or obstruction, and prior chemotherapy ≤ 6 months vs. >6 months. Kaplan-Meier curves and a two-tailed log-rank test will be the primary analysis methods. Assuming exponential survival curves, the hazard rate for the systemic therapy is 0.0231, or approximately a 2.3% probability of dying each month when the 5-year survival probability is 25%. If we assume that the MSLS arm will have an associated survival of 50% at 5 years, this survival corresponds to a hazard rate of 0.0116, and the resulting hazard ratio for the comparison of the two overall survival curves would be 1.991. Following the principles of a phase 2.5 design, to compare these curves and detect a difference with a 0.10 one-tailed log-rank test, a total of 35 evaluable subjects per arm (70 total) will need to be randomized over a five year period, followed for an additional two years from the date of entry of the last patient, and observation of 38 total deaths in order to have 80% power to compare the curves.

It is expected that 80% of registered patients will be eligible for the randomization. Thus, in order to enroll 70 randomized patients, a total of up to 100 evaluable patients may be enrolled. The patients who present prior to 11 months will be enrolled in the lead-in stage in which some patients may develop symptomatic or image-detected disease. These patients will not be eligible for randomization, but the number of randomized patients relative to enrolled patients will be noted in order to determine the denominator of patients who develop peritoneal recurrence within the first year of treatment. Registration will be permitted to continue until 70 patients have been randomized, and thus it is possible that up to 5-10 additional patients beyond 70 may be randomized. This will permit a very small increase in power. Patients will be analyzed as randomized; only patients found to be grossly inevaluable for any reason will not be included in the final analysis once randomized.

Progression-free survival will also be evaluated using Kaplan-Meier curves and a two-tailed log rank test, as a secondary endpoint. In addition, a prognostic factor evaluation using Cox proportional hazards modeling will take place after the study has concluded in order to identify if there are factors which can be identified that are associated with overall or progression-free survival in patients randomized to treatments on this trial; this analysis will be interpreted as a secondary endpoint.

It is expected that 20 patients per year can be accrued onto this trial, and thus accrual will be completed in approximately 5 years. Allowing for a very small number of inevaluable patients, the accrual ceiling will be set at 100 patients in order to permit 95 registrations and 70-75 randomized patients.

### Data and Safety Monitoring

Careful evaluation to ascertain the toxicity and clinical response will be performed. The principal investigator will monitor the data and toxicities in order to identify trends quarterly. The principal investigator will be responsible for revising the protocol as needed to maintain safety. The NCI IRB will review submitted adverse events monthly to evaluate trends and will require a follow-up plan from the principal investigator whenever a trend is identified.

The study will be monitored by the NCI/CCR Data Safety and Monitoring Board (DSMB) on an annual basis to evaluate the safety of the two arms. The serious adverse events (typically grade 3 or greater toxicities) will be reported according to type of toxicity, maximal grade noted per patient, and for toxicities with at least a possible attribution to the therapy provided on that arm. Comparisons will be made between the two arms using Cochran-Armitage tests for trend, or other appropriate methods, to determine if there is increased toxicity associated with either arm.

Beginning at the first annual DSMB meeting after 35 patients have been randomized, annual interim evaluations will be performed to determine if there is sufficient evidence to terminate accrual because of a better than expected improvement in survival.

### Inclusion and Exclusion Criteria

#### Inclusion Criteria

• Histologically confirmed colorectal adenocarcinoma.

• Curative resection (NED at closure) for CRC which was (1) perforated into the peritoneal cavity, (2) associated with minimal PC which was completely excised at the time of initial operation, (3) T4 lesion that required en bloc resection of additional organs, (4) associated with ovarian metastases or (5) emergency presentation with lesions associated with obstruction and/or bleeding.

◦ **Note**: Patient who presented at the time of diagnosis with limited extra abdominal metastases may be eligible if the lesions were completely resected and the patient remains NED.

• Received at least 3 months of standard of care adjuvant therapy and is disease free by conventional imaging at time of registration and randomization.

◦ **Note: **The imaging will be reviewed by an experienced radiologist and a surgical attending prior to enrollment.

• Greater than or equal to 18 years of age.

• Clinical performance status of ECOG ≤ 2.

• Stable serum CEA levels not indicating recurrence.

• No history of prior/other malignancies within the 2 years prior to enrollment with the exception of basal cell carcinoma.

#### Exclusion Criteria

• Active systemic infections, coagulation disorders, or other major medical illnesses precluding major surgery.

• Prior experimental therapy with novel agents.

• Prior HIPEC.

• History of brain metastases.

• Childs B or C cirrhosis or with evidence of severe portal hypertension by history, endoscopy, or radiologic studies or with evidence of moderate to severe ascites.

• Weight less than 40 kg.

• History of congestive heart failure and/or an LVEF < 40%.

• Significant COPD or other chronic pulmonary restrictive disease with PFT's indicating an FEV1 less than 50% or a DLCO less than 40% predicted for age.

### Intervention

#### Mandatory Second-Look Surgery (MSLS) Arm

All patients randomized to the MSLS arm will undergo exploratory laparotomy at the Surgery Branch, NCI. The overall goal for surgical therapy is to determine whether clinically occult metastases exist and to render the patient NED with negative margins if metastases are detected. All previous dissection planes should be open as feasibly and safely as possible which requires an exploratory laparotomy because most recurrences detected at sites of prior operative therapy that often have adhesions preventing laparoscopic evaluation of these areas. Determination of NED status will be based upon visual inspection, frozen biopsy of any suspicious lesions, and ultrasound of liver with biopsy as indicated. Peritoneal washings will be performed in all patients. Sixteen16 peritoneal biopsies will be done - 4 from each quadrant. Data from these investigations will be used at the final analysis of the risks and benefits of this approach. Pre- and post-operative peritoneal disease will be recorded using the PCI and the Gilly's methodology [35,36]. If any means above detect recurrent disease, all disease will be resected if technically feasible and HIPEC will be performed in all patients. Complete peritonectomy will be performed only in cases where carcinomatosis is detected and confirmed by a frozen biopsy. For patients with peritoneal disease peritonectomy and CRS includes the following: The right and left sub-diaphragmatic peritoneum, the falciform ligament, lesser and greater omentum, anterior, right and left abdominal wall down to the paracolic gutters, and the pelvic peritoneum. The surgeon has the discretion to perform partial peritonectomy for limited disease which may include one or more of the resections described above. Intra-operative hepatic ultrasound will be performed when clinically indicated.

For the HIPEC procedure, two large bore catheters (thoracostomy tubes) will be inserted through the abdominal wall incision, one over the right lobe of the liver and one in the pelvis. The abdominal skin will be closed and the catheters connected to a perfusion circuit. The perfusate passes from a reservoir through a roller pump, heat exchanger, and then into the abdominal cavity. Efflux from a second catheter is then recirculated through the reservoir and pump. The perfusion flow rate will be maintained at 2.0 L/min and a perfusate volume will be maintained which moderately distends the abdominal cavity correlating with intra abdominal pressures of 5 to 15 mm Hg (2.0 L/m^2^). Stable perfusion parameters are obtained and the peritoneal cavity is warmed to a minimum of 41°C prior to starting the clock for perfusion time and a maximum of 43°C. The perfusion will be continued for 30-35 minutes. During the perfusion, constant physical manipulation of the abdomen (shaking) will be maintained for the entire perfusion period to assure even distribution of the perfusate. The heater coil will be maintained at 46-48°C. Peritoneal temperature will be measured continuously by four probes placed immediately beneath the peritoneal surface on either side of the abdomen and in the pelvis. The patient's core temperature will be measured with an esophageal probe (which correlates well with pulmonary artery temperatures) and maintained at less than 41°C using a cooling blanket and ice packs around the legs and head. At the end of the perfusion, the abdomen will be re-opened and the perfusate irrigated from the abdominal cavity. All intraperitoneal drug dosages will be calculated on ideal body weight. 5-FU (400 mg/m^2^) and leucovorin (20 mg/m^2^) will be administered intravenously 15 minutes prior to HIPEC to potentiate the activity of oxaliplatin (460 mg/m^2 ^diluted in 2.0 L/m^2 ^of D5W via the perfusion circuit).

During the post-operative period, patients will receive all standard of care supportive measures. For patients who undergo HIPEC, these measure include when indicated: Broad-spectrum antibiotics for neutropenic fever; filgrastim for ANC less than 1000 mm^3^, and avoidance of renal toxic drugs, maintenance of good renal perfusion, and diuretics as necessary for renal dysfunction.

#### Standard of Care Surveillance (SC) Arm

Patients in both arms will undergo the same follow-up surveillance as noted in the next section. Patients who have peritoneal recurrences detected on the surveillance arm will be offered exploration if clinically appropriate with potential CRS and HIPEC pending intra-operative assessment of extent of disease.

#### Post-randomization surveillance for both arms

Follow-up will commence from randomization and will occur every 3 months for two years. After two years, follow-up will be extended to every six months, and after an additional three years, follow-up will be yearly. At any stage between these follow-ups additional evaluations will be conducted as clinically indicated. At each evaluation patients will undergo: Physical examination; laboratory tests; tumor markers; CT scan of the chest, abdomen and pelvis; PET as medically indicated. Patients on both arms who develop intra/extra-abdominal metastases will be offered standard of care surgery when indicated and/or referred to their treating physician or Medical Oncology Branch, NCI, for systemic therapy.

### Informed Consent

All patients are thoroughly screened prior to initial consultation at the NIH. During the initial consultation, the patient, along with family members, is presented a forthright and detailed overview of the treatment option available to them at the NIH. The experimental nature of the treatment, its theoretical advantages and disadvantages, and an overview of the operative procedure and anticipated convalescence are presented. The fact that the patient may undergo an operative procedure in order to receive therapy without any assurance of benefit, the aggressive nature of the treatment, and the possibility of serious or potentially life-threatening complications are presented. The Informed Consent document is given to the patient and they are asked to review it, make notes and follow-up with a phone call to the physician or nurse investigator to have any additional questions answered prior to considering treatment on protocol. The research nurse, principal investigator, or designee is responsible for obtaining consent from the patient upon admission. The signed consent will be verified by the physician responsible for the care of the patient. Patients can withdraw or decide against treatment at any time without obligation.

### Endpoints and Follow-up of Study

#### Primary Objectives

• To compare the overall survival of patients at high risk for developing PC from CRC who undergo MSLS with HIPEC and CRS (if applicable) vs. similar patients who receive standard of care surveillance.

#### Secondary Objectives

• To determine recurrence-free survival from the time of randomization in both arms.

• To investigate selection criteria for patients who might benefit from MSLS with HIPEC and CRC.

• To determine the percentage of patients who are diagnosed with recurrent peritoneal disease (gross vs. microscopic) at exploration.

• To determine the percentage of patients with recurrent disease at MSLS who are successfully debulked to R0.

• To determine the rate of clinical detection of PC during the pre-randomization period and thereby determining the denominator for this cohort of patients.

## Discussion

Colorectal cancer is the 3^rd ^most common malignancy in the United States accounting for over 100,000 new cases per year [1]. Peritoneal carcinomatosis accounts for about 13% of metastatic spread among patients who die from this disease [2]. Despite significant advances in chemotherapy with regimens such as FOLFOX and FOLFIRI, at best about 13% of patients with peritoneal carcinomatosis achieve 5-year survival with chemotherapy alone [26]. CRS and HIPEC have shown impressive results with 5-year survivals of about 50% for patients with disease limited to the peritoneum [11,13,23]. The patients who consistently experience the best survivals undergo complete resection of peritoneal disease (R0 resection). Unfortunately, this disease is not detectable by imaging, symptoms, or tumor markers early in the disease, yet early intervention is more likely to achieve complete resection of the disease. Therefore, identification of a patient population at high risk for PC may allow earlier interventionin order to undergo surgery completely resect recurrent disease has been proposed to increase survival in this patient population.

This trial tests the strategy of mandatory second-look laparotomy for patients at high risk of recurrent peritoneal carcinomatosis versus the current standard-of-care of routine surveillance. The high risk patients previously have been defined as having limited and resected peritoneal disease at initial operation, ovarian metastases, tumor perforation, T4 lesions with en bloc adjacent organ resection, and emergency presentation with bleeding and obstruction. Given that this patient population has high risk of peritoneal recurrence that translates into very poor survival, a strategy as invasive as an mandatory exploratory second-look surgery is justified to determine its impact on survival, yet this proposal should be subjected to rigorous scientific scrutiny prior to implementation outside of a clinical trial. If the primary objective of increasing overall survival is met, this trial may change the current management of patients at high risk for developing recurrent peritoneal carcinomatosis to one of aggressive, early, surgical intervention with strong evidence to show that patients will benefit from this strategy. Hopefully, this treatment will significantly increase long-term survival among patients with peritoneal carcinomatosis.

## Completing interests

The authors declare that they have no competing interests.

## Authors' contributions

IA is the principal investigator for the study described in the manuscript. RTR and IA are responsible for the concept and design. RTR, JLD, CDK, SMS, MAT, and IA made significant contributions to protocol validity, design, drafting, and revising of the manuscript. SMS developed the statistical considerations for the trial. RTR, JLD, CDK, SMS, MAT, and IA contributed to the scientific accuracy of the manuscript. IA gave the final approval for the final version to be published.
